# Impact of Hanks Kinase‐Dependent Phosphorylation of CodY on the Physiology and Virulence in *Bacillus cereus*

**DOI:** 10.1002/mbo3.70103

**Published:** 2025-11-04

**Authors:** Mounia Kortebi, Céline Henry, Christophe Buisson, Christelle Lemy, Michel Gohar, Didier Lereclus, Ivan Mijakovic, Sandrine Poncet

**Affiliations:** ^1^ Institute for Integrative Biology of the Cell (I2BC) CEA, CNRS Université Paris‐Saclay Gif sur Yvette France; ^2^ INRAE, AgroParisTech, Micalis Institute Université Paris‐Saclay France; ^3^ Systems and Synthetic Biology Chalmers University of Technology Göteborg Sweden; ^4^ Novo Nordisk Foundation Center for Biosustainability Technical University of Denmark Kgs. Lyngby Denmark; ^5^ UMR408 Sécurité et Qualité des Produits d'Origine Végétale INRAE, Avignon Université Avignon France

**Keywords:** *Bacillus cereus*, CodY, differenciation, Ser/Thr kinase, transcriptional regulation, virulence

## Abstract

CodY acts as a key regulatory protein involved in adaptive responses in low‐G+C Gram‐positive bacteria. This global transcriptional regulator diagnoses the nutritional status of the cell and responds by regulating transcription of genes involved in metabolism, differenciation and virulence. Phosphoproteomic studies evidenced that CodY is phosphorylated on its serine 215 in *Bacillus subtilis*. In *Bacillus cereus*, CodY is also phosphorylated by the Hanks kinases PrkC and YbdM. CodY phosphorylation negatively affects its DNA‐binding properties. We constructed *B. cereus* mutant strains where the *codY* wild‐type allele has been replaced by *codY*‐S215D or *codY*‐S215A, encoding a phosphomimetic or a phosphoablative CodY derivative, respectively. We showed that the phosphomimetic mutation leads to a notable reduction in CodY control over several critical cellular processes, including motility, biofilm formation, cytotoxic effects and pathogenicity. Lack of CodY phosphorylation and CodY overphosphorylation have opposite repercussions on gene expression, showing that CodY phosphorylation contributes to the adaptation of *B. cereus* to diverse environmental conditions. S215 is strictly conserved in CodY orthologs in firmicutes, suggesting that gene regulation mediated by Hanks kinase‐dependent CodY phosphorylation could be a general regulatory mechanism in this phylum.

## Graphical abstract

CodY is a global regulator whose phosphorylation at serine 215 by PrkC/YbdM alters its DNA binding, thereby modulating gene expression and impacting motility, biofilm formation, cytotoxicity, and pathogenicity in *Bacillus cereus*, suggesting a conserved regulatory mechanism in Firmicutes

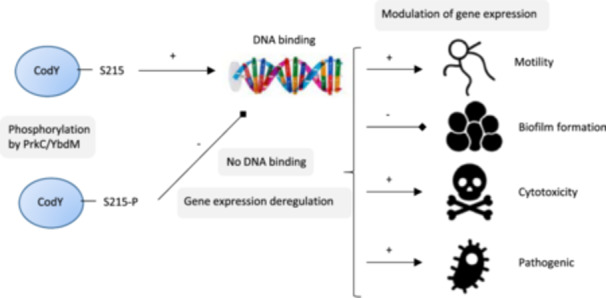

## 1 Introduction

Pathogenic bacteria need to adapt to various conditions and to resist stresses encountered during the infection process. To adapt effectively, bacteria rely on global regulatory systems capable of adjusting differentiation pathways in response to a broad spectrum of environmental signals. Signals are frequently transmitted through protein phosphorylation (Gangwal et al. 2023). Recent phosphoproteomic studies pointed out the role of bacterial tyrosine kinases (BYs) (Grangeasse et al. 2007; Grangeasse et al. 2012) and Hanks‐type serine/threonine protein kinases (STKs) and their cognate phosphatases (STPs) in these signaling networks (Stancik et al. 2018; Zhang 1996; Kobir et al. 2011; Mijakovic and Macek 2012; Roumezi et al. 2020). In pathogenic Gram‐positive bacteria such as *Staphylococcus aureus*, *Mycobacterium tuberculosis* and Streptococci, STKs and STPs are frequently involved in virulence and infection (Fridman et al. 2013; Wright and Ulijasz 2014; Pensinger et al. 2018; Ren et al. 2022). As a result of their relaxed specificity, STKs constitute nodes in the regulatory networks. STKs phosphorylate a diversity of protein targets, including transcriptional regulators like Sigma factors and RNA‐polymerase (RNAP) subunits, and provide an additional mean by which bacteria modulate gene transcription (Cousin et al. 2013; Wright and Ulijasz 2014; Kalantari et al. 2015; Garcia‐Garcia et al. 2016). Phosphorylation of a transcriptional regulator can affect its recruitment by σ‐factors and/or RNAP or impedes its DNA binding by either preventing the oligomerization of sister subunits or directly inhibiting the hydrogen‐bonding to target DNA. Nevertheless, only a limited number of studies investigate the effects of Ser/Thr phosphorylation on transcriptional regulators that govern stationary phase processes in spore‐forming Firmicutes. *Bacillus subtilis*, the model and most extensively studied sporulating firmicute, encodes three STKs, PrkC, YbdM, and YabT. These kinases are expressed during the transition and stationary phase, when genes are subjected to multiple forms of regulation, depending on nutrient availability. Nutrient depletion triggers the activation of stationary phase genes associated with differentiation pathways: biofilm formation, sporulation or competence. PrkC, whose extracellular domain senses muropeptides, is involved in stationary phase survival, sporulation, germination and biofilm formation but also in the control of cell‐wall metabolism (Madec et al. 2002; Shah and Dworkin 2010; Libby et al. 2015; Pompeo et al. 2016). YbdM intervenes in competence and swarming (Jers et al. 2011) and YabT sustaine chromosome integrity during the process of sporogenesis (Bidnenko et al. 2013) and regulating sporulation and biofilm development (García García et al. 2018). AbrB and CodY, two pleiotropic transcriptional regulators governing the expression of stationary phase functions, were identified by phosphoproteomic studies as putative STKs targets (Macek et al. 2007; Soufi et al. 2010). AbrB and CodY are phosphorylated in vivo on their residues serine 86 and 215, respectively. AbrB Ser86 is positioned near its C‐terminal domain responsible for oligomerization. Its STK‐dependent phosphorylation induces a conformational change that interferes with its ability to bind DNA, resulting in the dysregulation of numerous downstream target genes and affects key target functions in *B. subtilis*: exoprotease production, competence development and sporulation (Kobir et al. 2014). CodY was initially discovered in *B. subtilis* as a negative regulator of the dipeptide permease operon (Slack et al. 1995), and later identified as an essential element governing the expression of genes related to the stationary phase and spore formation (Molle et al. 2003). CodY turned out to be a pleiotropic transcriptional regulator present in all firmicutes, which mostly represses stationary phase genes during the exponential growth by sensing the intracellular pool of BCAA (Branched‐chain amino acids) and GTP (Ratnayake‐Lecamwasam et al. 2001; Shivers and Sonenshein 2004). Binding of BCAA and GTP to CodY N‐terminal domain induces conformational changes and activates its DNA binding properties. CodY thereby represses the transcription of numerous genes. CodY binding to promoter regions requires the recognition of specific “CodY boxes” by its C‐terminal domain, which adopts a helix‐turn‐helix motif. Interestingly, this HTH region is highly conserved in firmicutes, and the amino acid located at position 215 is always a serine (Joseph et al. 2005). In *B. subtilis*, CodY regulates more than 200 genes and influences key stationary phase survival functions such as competence, exoprotease production, biofilm formation and sporulation (Brinsmade et al. 2014). CodY also links virulence to metabolism in many low G + C Gram positive bacteria (Sonenshein 2005; van Schaik et al. 2009, Dineen et al. 2010; Stenz et al. 2011; Richardson et al. 2015). Interestingly, global phosphoproteomic studies evidenced that in *B. subtilis*, CodY is phosphorylated on its S215 residue (Macek et al. 2007).

Members of the *Bacillus cereus sensu* lato group (including *B. anthracis*) include *Bacillus* species with pathogenic potential (Ehling‐Schulz et al. 2019), which share with *B. subtilis* a substantial set of transcriptional factors that regulate stationary phase processes, such as the sporulation regulator Spo0A, the sigma factor σ^H^, the phase‐transition regulators SinR, AbrB and CodY. All these regulators are involved in the control of biofilm formation, virulence and necrotrophism in bacteria of the *B. cereus* group (van Schaik et al. 2009; Château et al. 2011; Frenzel et al. 2012; Lindbäck et al. 2012; Dubois et al. 2013; Fagerlund et al. 2014; Slamti et al. 2015). *B. cereus* encodes two Hanks kinases, which are orthologs to *B. subtilis* PrkC and YbdM (BC3860 and BC2258, respectively) and two serine/threonine phosphatases PrpN (Gangwal et al. 2022; BC2070 in *B. cereus* ATCC 14579) and PrpC (BC3861, which gene is co‐transcribed with *prkC* (Kortebi and Poncet, unpublished results)). PrkC is involved in virulence, biofilm formation and spore germination in *B. anthracis* (Arora et al. 2017; Dhasmana et al. 2021; Gangwal et al. 2022), while no role has been assigned to YbdM in bacteria of the *B. cereus* group. In *B. anthracis*, CodY is phosphorylated by PrkC on its Serine 215 residue, and CodY‐S215‐P is dephosphorylated by PrpN, a ser/thr phosphatase. CodY‐S215D, a phosphomimetic variant of CodY, cannot bind to the *atxA* promoter, which encodes the global virulence gene regulator AtxA (Gangwal et al. 2022). In this study, we examined in *B. cereus* strain ATCC14579, the impact of CodY S215 phosphorylation on its regulatory activity and on the phenotypes it controls.

## 2 Results and Discussion

### 2.1 CodY Is Phosphorylated by Hanks‐Type Kinases in *Bacillus cereus*

**Figure 1:**
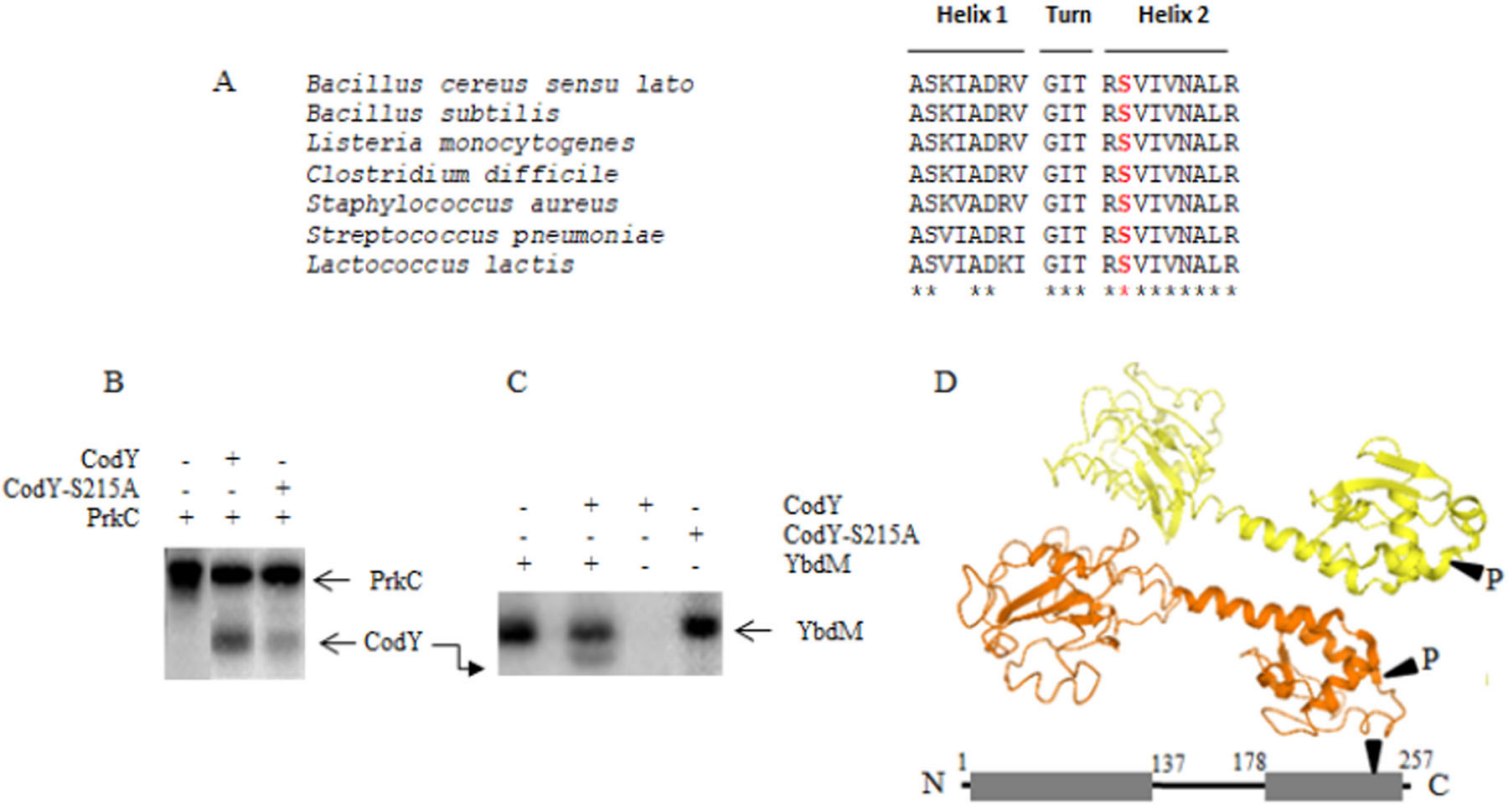
In vitro phosphorylation of CodY by Hanks kinases. (A) Alignment of *B. cereus* CodY with CodY homologs of a variety of firmicutes. Residues 203 to 222, covering the HTH domain are represented. The three‐dimensional structure of CodY (Bacillus subtilis) was obtained from the Protein Data Bank (PDB ID: 5J8F) (Levdikov et al. 2006). (B) In vitro phosphorylation assays of CodY and CodY‐S215A (non‐phosphorylatable) in the presence of PrkC and (B) in the presence of YbdM. (C). Presence of proteins is indicated with +/− above each line. Radioactive bands corresponding to sizes of phosphorylated proteins are indicated by arrows. (D) Mapping of the CodY S215 phosphosite within the HTH region and CodY dimer model showing the S215 sites susceptible to phosphorylation.

In *B. anthracis* as in *B. subtilis*, CodY is phosphorylated on its ser215 residue, which drastically inhibits its binding to target promoter (Joseph et al. 2005; Gangwal et al. 2022). S215 is strictly conserved among CodY orthologs, including those of other members of the *B. cereus* group and seems to serve as a pivotal component in CodY‐mediated regulation (Figure 1A). We hypothesized that CodY might also be phosphorylated in *B. cereus* ATCC 14579 by STKs and that this modification may impact the expression of CodY‐dependent adaptative and virulence genes. To test whether PrkC and YbdM can phosphorylate CodY in vitro, *B. cereus* CodY and its non‐phosphorylatable version CodY‐S215A, PrkC (cytosolic catalytic domain) and YbdM were overexpressed in *E. coli* as N‐terminus (His)_6_‐tagged proteins and purified as described in experimental procedures (Supporting Information S1: Figure S1A). CodY was then incubated in the presence of *B. cereus* PrkC (Figure 1B) or YbdM (Figure 1C), and both STKs were able to phosphorylate it. We also tested the autophosphorylation of CodY: after an incubation for 1 h in the presence of ATP 50 μM, no autophosphorylated CodY was evidenced (Figure 1C). Our results differ from those of Joon et al. who showed that CodY from *B. anthracis* was able to autophosphorylate in a GTP‐ and to a lesser extent in an ATP‐dependent manner (Joon et al. 2017). When the CodY‐S215A (Alanine being a non phosphorytable amino acid) was used as a substrate, the phosphorylation diminished considerably (PrkC) or was abolished (YbdM), suggesting that the STK‐dependent phosphorylation of CodY concerns S215 (Figure 1B,C, right panel). One may therefore propose that in *B. cereus* ATCC 14579 both YbdM and PrkC are able to phosphorylate CodY on its serine 215 residue. In *B. anthracis*, PrkC has been shown to phosphorylate CodY (Gangwal et al. 2022). However, the authors did not explore the role of YbdM in CodY phosphorylation.

### 2.2 CodY Phosphorylation Impedes Its DNA Binding

The interaction between CodY and DNA relies on the presence of a specific sequence (the CodY box) found within the promoter regions of target genes. Ser 215 maps in the HTH domain of CodY (residues 203–222), which is highly conserved among firmicutes (Figure 1A). In all CodY orthologs, Ser215 is an invariant residue located in the turn between α7 and α8 (which correspond to Helix1 and 2 of the HTH domain); Ser215 lies on the face of the HTH domain, which corresponds to the DNA‐binding interface (Joseph et al. 2005; Levdikov et al. 2017 *;* Gangwal et al. 2022. Figure 1D). In vitro, affinity of *B. subtilis* ‐S215T, ‐S215F, and –S215A CodY variants for known target promoters is reduced, and repression of these promoters in corresponding *codY* mutant strains is weaker than in the wild‐type strain (Joseph et al. 2005). *B. anthracis* and *B. cereus* ATCC14579 CodY are 99.6% similar, with the HTH domain fully conserved. We hypothesize that introducting a negative charge at S215, either by phosphorylation or by phosphomimetic mutation (CodY‐S215D), induces electrostatic repulsion with the DNA, causing loss of DNA/protein interaction. We therefore anticipated that in *B. cereus* ATCC14579, DNA‐binding by CodY may be negatively affected by S215 eSTKs‐dependent phosphorylation. We conducted gel shift assays using a promoter region directly regulated by CodY. Complete phosphorylation of proteins by bacterial is seldom attainable in vitro. We then used point mutants CodY‐S215A and CodY‐S215D to mimic non‐phosphorylatable and fully phosphorylated state of CodY, respectively. We first verified that point mutations did not disrupt CodY overall structure. CodY, CodY‐S215A and ‐S215D structural integrity was evaluated using circular dichroism (CD) spectroscopy (Supporting Information S1: Figure S1B). The spectrum of each variant displayed the characteristic pattern of a folded α‐helical protein, comparable to that of the wild‐type CodY, indicating that S215A and S215D mutations did not alter the overall protein structure. Gel filtration analysis (Supporting Information S1: Figure S1D) showed that the elution volumes of CodY and of its phospho‐ablative and phospho‐mimetic variants were all three identical and corresponded to the elution volume of a CodY dimer (Monomer Mw = 29.8 kDa). Our results differ from previous data, which suggested that in absence of GTP and BCAA, *B. cereus* CodY adopts a tetrameric inactive state (Han et al. 2016).

**Figure 2:**
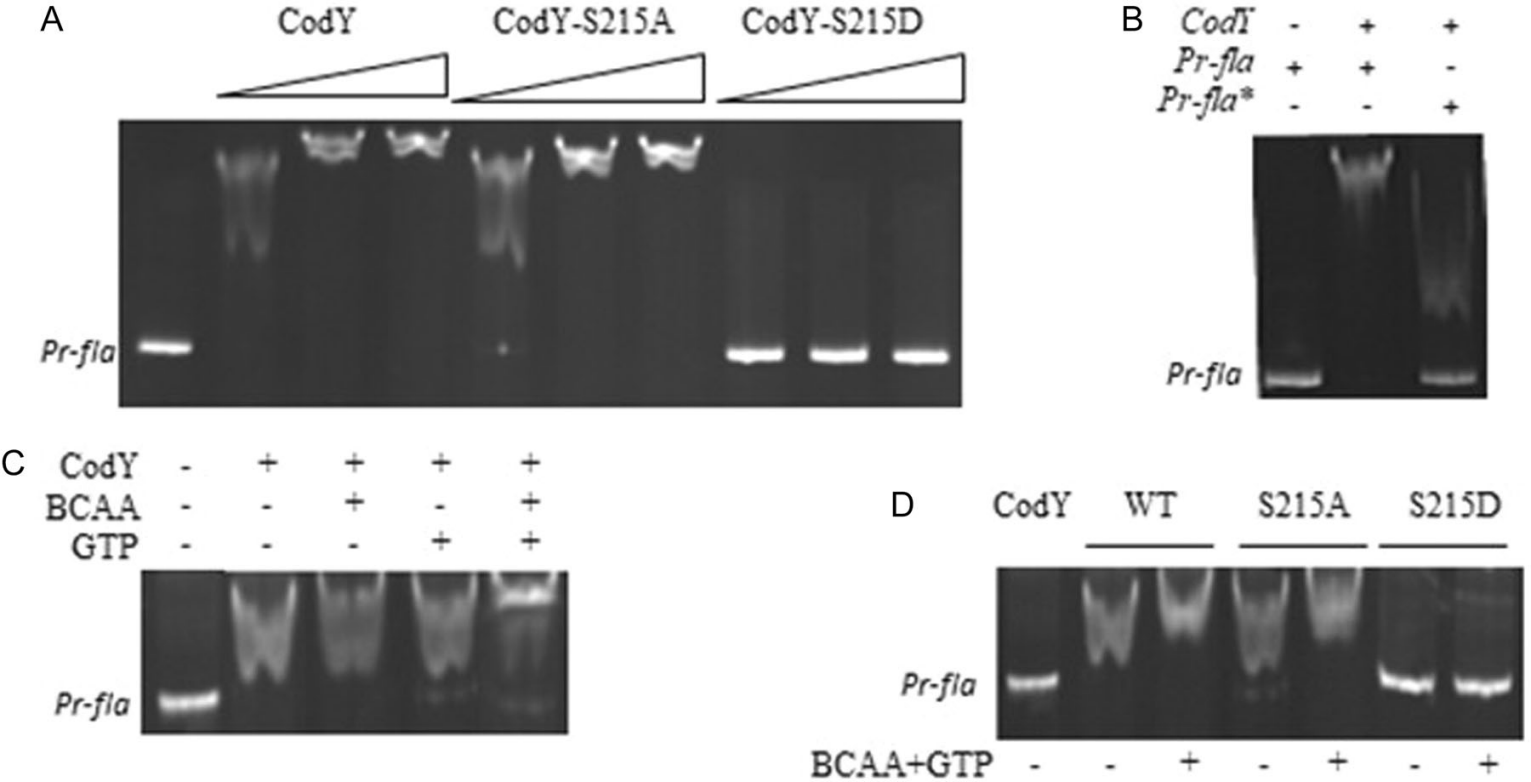
Specific binding of CodY and its phosphoablative and phosphomimetic versions to the *fla* operon promoter region. (A) Binding of CodY and its mutant versions to a 151 bp fragment encompassing the CodY box located in the promoter region of the *fla* operon was assayed by gel shift. The assay contained 40 nM target DNA and 2, 4, and 6 μM of either CodY WT, CodY‐S215A, and CodY‐S215D. (B) 4 μM CodY were incubated in the presence of 40 nM of the 151 bp *fla* region (left) or 40 nM of the same region harboring a mutated CodY box (fla*, right). (C) Synergistic effect of GTP and BCAA. The concentration of target DNA (40 nM) and CodY (2 μM) was kept constant, and BCAA 10 mM and GTP 2 mM were added when indicated. Samples were loaded on the gel after 20 min of incubation at room temperature. (D) 40 nM of target DNA incubated with 2 μM of CodY or its derivatives, in the absence (−) or the presence (+) of BCAA+GTP (10 and 2 mM, respectively).

Expression of the flagellar operon BC1657 to BC1659 in *B. cereus* strain ATCC14579 is dramatically impeded (12.5‐ and 25‐fold, respectively) in a Δ*codY* background (Lindbäck et al. 2012), inactivation of *codY* leading to an absence of cell motility. CodY therefore acts as a positive regulator of flagellar gene expression. Chateau and collaborators identified, using a genome‐wide approach, a CodY consensus motif in *B. anthracis* (Chateau et al. 2013). A BLAST analysis evidenced the presence of a putative *B. anthracis*‐like CodY box (Supporting Information S1: Figure S2A) in the promoter region of the flagellin operon. Extending this sequence to upstream nucleotides (AATTAACAGAAAAAT) provides a binding site corresponding to the specific weight matrix of CodY‐binding motifs previously defined in *B. subtilis* (Belitsky and Sonenshein 2008). Surprinsingly, this box is located between the putative ‐35 (TTAACA) and ‐10 (TATAAA) sequences separated by 17 nucleotides (Supporting Information S1: Figure S2B), what comes close to *B. subtilis* consensus sequences recognized by σA (TTGACA‐17nt‐TATAAT) (Haldenwang 1995). CodY binding site then overlaps this putative promoter, and CodY binding will then likely inhibit the transcription initiation rather than promote the expression of the flagellar operon, as deduced by the global transcriptional analysis of Lindback and coll. (Lindbäck et al. 2012). We PCR‐amplified a 151 bp fragment (using oligonucleotides SAT201 and SAT202, Supporting Information S1: Table S1) encompassing these sequences (Supporting Information S1: Figure S2B) and compared the binding of CodY and its phospho‐ablative (CodY‐S215A) and phospho‐mimetic (CodY‐S215D) variants (Figure 2A). In the assays, DNA concentration was held constant while the amount of CodY was varied. Wild‐type CodY bound to the DNA probe (Pr‐*fla*) and a high molecular weight complexe was formed. The CodY‐S215A variant bound DNA with comparable efficiency. By contrast, CodY‐S215D lost its binding capacity, even at high concentration. However, mutation of the CodY consensus box (Pr‐fla*) abolished the binding of CodY (Supporting Information S1: Figure S2 and 2B). We also examined the effect of GTP and BCAA (Figure 2C). GTP and BCAA had a synergistic action on CodY binding (Figure 2C,D). CodY‐S215A binding was also strengthened in the presence of GTP and BCAAs, whereas CodY‐S215D remained unable to bind DNA (Figure 2D). From these data we concluded that CodY S215 phosphorylation drastically attenuates its affinity for DNA, whereas a phosphoablative version of CodY retains full capacity to bind DNA and to bind both BCAA and GTP. These results suggest that YbdM‐ and PrkC‐dependent phosphorylation represents a new mechanism in CodY‐dependent gene regulation, as suggested in previous studies (Joseph et al. 2005; Gangwal et al. 2022).

## 3 Physiological and Adaptative Effects of CodY Phosphorylation

Given the wide spectrum of activity of known Hanks kinases in Gram‐positive bacteria, we hypothesized that in *B. cereus* also, PrkC and YbdM phosphorylate a broad range of substrates, making in vivo analyses especially challenging. For further studies, ATCC14579 point mutants *codY‐*S215A and *codY‐*S215D were therefore constructed, to mimic the expression of CodY in its non phosphorylatable or fully phosphorylated state, respectively. For that purpose, we used the ATCC14579 Δ*codY* mutant strain constructed by Lindback et al. a seamless deletion strain with no polar effect (Lindbäck et al. 2012). Using pMad‐derived vectors (pMad‐*codY*‐S215A and pMad‐*codY*‐S215D), we reintroduced at the *codY* locus of this strain either the *codY*‐S215A or the *codY*‐S215D allele by homologous recombination (See Experimental procedures). The presence and precise location of the intended mutations were verified by amplifying the *codY* region from the chromosome and sequencing the resulting PCR products. We performed a Western blot analysis assay (Supporting Information S1: Figure S3) and showed that the CodY variants were detected in equivalent amount in all strains, indicating that point mutations affect neither the accumulation nor the stability of CodY in vivo. However, CodY concentration in the three strains remained constant whatever the growth phase was, as previously demonstrated (Ratnayake‐Lecamwasam et al. 2001).

Next, we aimed to confirm our hypothesis that phosphorylation of CodY is an effective inactivation mechanism in vivo. In *B. cereus*, CodY controls the expression of genes involved in key stationary phase functions: CodY represses genes involved in biofilm formation and activates those involved in mobility and virulence, but is also required for good fitness in various rich medium (Frenzel et al. 2012; Kovács 2016; Lindbäck et al. 2012; Slamti et al. 2016). To investigate the consequences of CodY phosphorylation in *B. cereus*, we examined these physiological processes and the expression profile of associated promoters for which the role of CodY has been established.

### 3.1 CodY Phosphorylation Participates in Cell Growth and Global Protein Expression Pattern

**Figure 3:**
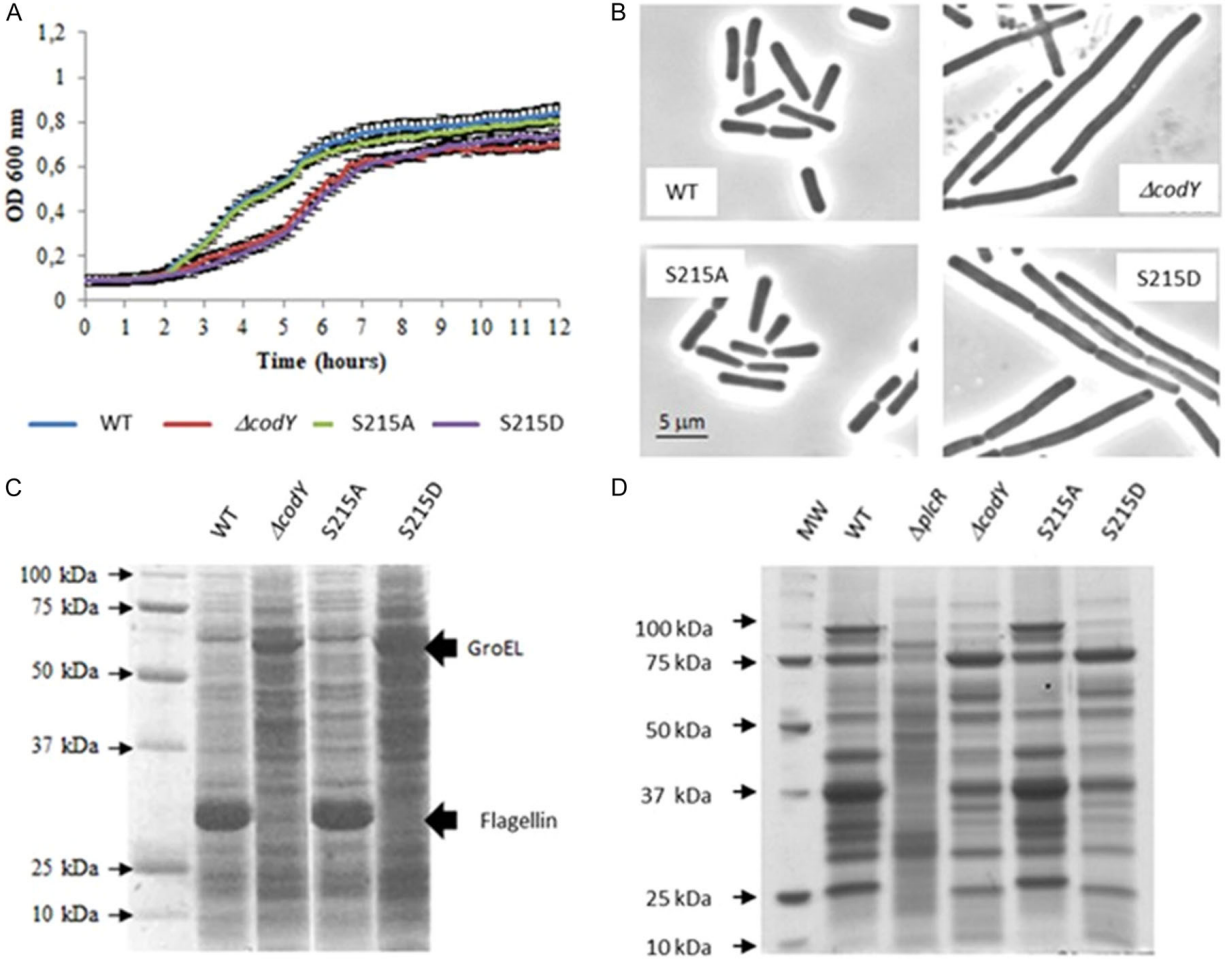
CodY phosphorylation affects cell growth, cell morphology and global protein expression. (A) Strains were grown in BHI at 37°C in a microplaque reader (TECAN). Data are averages of two independent experiments (error bars are SEM from mean values). (B) Samples were taken at OD600 0,4 and examined by phase contrast microscopy. C) 200 ul aliquotes were taken 2 hours after t0 and concentrated 10 fold in SDS sample buffer. Proteins were then separated by SDS‐PAGE on a 15% polyacrylamide gel. (D) 2 mg of proteins from culture supernatants were separated by SDS‐PAGE on a 12.5% polyacrylamide gel. Samples were taken 2 hours after t0. Immediately after harvesting, the culture supernatants were centrifuged at 8000 rpm for 20 min at 4°C. The supernatant of the centrifugation was rapidly filtered through a membrane (pore size = 0.2 mm; Nalgene Sterilization Unit, Nalge Company). Proteins were then precipitated twice using the deoxycholate‐tetrachloroacetic acid method (Peterson 1983). The pellet was washed twice with ethanol:ether (1:1), and dried and stored at −80°C until use. The protein content of the pellet was determined by the Bradford method.

Changes in cell morphology have been previously observed depending on the presence of CodY: Whereas the growing wild‐type culture contained individual cells, the Δ*codY* mutant strain displayed extensive cell chaining (Lindbäck et al. 2012). When grown in rich medium, the S215A variant behaved like the WT strain (Figure 3A,B), whereas the S215D variant behaved like the Δ*codY* strain, suggesting that CodY‐S215A is fully able to fulfill its physiological role whereas CodY‐S215D is not. This result, even if the OD based determination might be biased by the altered cell morphology, reinforces the idea that CodY phosphorylation participates in cellular growth and division, as previously described (Huillet et al. 2017). In addition, the comparison of the cellular and extracellular protein profiles (Figure 3C,D, respectively) revealed major differences between the WT and S215A strains on the one hand, and Δ*codY* and S215D strains on the other hand. The major protein accumulated in growing WT and S215A cells and severely decreased in strains Δ*codY* and S215D was identified by LC‐MS/MS analysis as BC1659, a flagellin essential for cell motility. By contrast, the major protein accumulated in Δ*codY* and S215D was identified as GroEL, which is one of the 100 most abundant proteins in *B. subtilis*. GroEL is an essential molecular chaperone involved in bacterial protein homeostasis mechanisms, which expression is induces in a large set of stress conditions (Nicolas et al. 2012). Accumulation of GroEL in strains *codY* and S215D may therefore reflect the stress encountered by the bacteria when CodY is unable to fulfill its physiological role. Major extracellular proteins, including virulence factors, are positively regulated by PlcR in B. cereus and are no longer detectable when *plcR* is inactivated (*plcR* strain, Figure 3D). As a key transcriptional regulator, PlcR facilitates the adaptive response of *Bacillus cereus* to environmental changes (Gohar et al. 2008), and its activity is controlled by CodY through its role in the reimport of its cognate signaling peptide PapR (Slamti et al. 2016). We examined by SDS‐PAGE followed by Coomassie blue stain the main extracellular proteins from strains WT, Δ*codY*, S215A and S215D (Figure 3D). Once again, we found that the exoprotein profile of strain S215A was close to that of the WT strain, while that of both the Δ*codY* and S215D strains was distinct. The exoprotein profile of the Δ*codY* and S215D strains is however different from that of the *plcR* strain, which suggests that production of major extracellular proteins is regulated by CodY (Barbieri et al. 2016), but also by additional pathways in *B. cereus*.

### 3.2 CodY Phosphorylation Antagonizes Flagellum‐Dependent Motility

**Figure 4:**
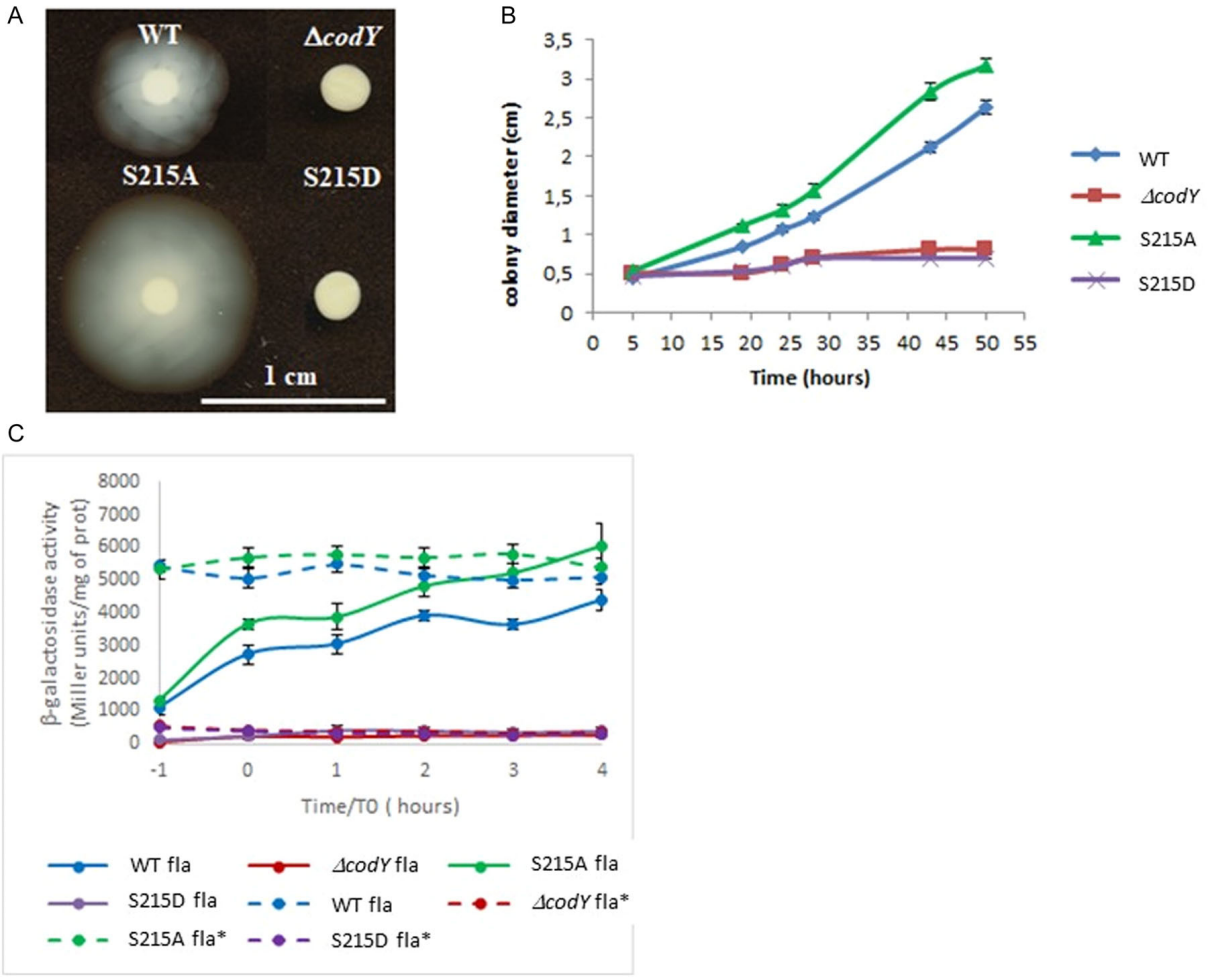
Swimming is inhibited by CodY phosphorylation. (A) 0.2% agar plates were inoculated with the wild‐type or the mutant strains and incubated 19 hours at room temperature. The results shown here are representative samples from two biological replicates. (B) Swimming velocity of the wild‐type and mutant strains. 5 ml of vegetative cells were spotted on 0,2% agar plates and incubated at room temperature. Diameters of the colonies were plotted against the incubation time. The results are the mean values of two independent experiments with 3 clones and the error bars represent SEM. (C) Activity of the *fla*‐ and fla*‐*lacZ* transcriptional fusions in the WT, codY, S215A and S215D backgrounds. Strains were grown in LB at 37°C and *t*0 designates the end of the exponential phase. Data are averages of three independent experiments (error bars are SEM from mean values).

As previously discussed, CodY acts as a positive regulator of *fla* operon expression in *B. cereus* ATCC14579 (Lindbäck et al. 2012). Moreover, we demonstrated that both CodY and CodY‐S215A are able to bind to the CodY box of the *fla* promoter region while CodY‐S215D is not (see above). We examined in vivo the influence of S215 mutations on the motility of *B. cereus* by plating the WT, *codY*, S215A and S215D strains on LB supplemented with 0.2% agar. After 19 h at room temperature, the motility of the S215A strain was greater than for the WT strain, and almost inexistant in strains ∆*codY* and S215D (Figure 4A). The rate of colony development on soft agar was higher for strain S215A than for the WT strain, while it was almost null in both the Δ*codY* and S215D strains (Figure 4B). To verify if differences in flagellin gene transcription could explain the differences of motility between these strains, they were transformed with pHT18Z‐*fla' or p*HT18Z‐fla*, carrying the wild‐type *fla* promoter region or that comprising the mutated *codY* box used in gel retardation experiments, respectively (see Supporting Information S1: Figure S2). Expression of the *fla* operon is induced in early stationary phase in *B. thuringiensis* Bt407‐ (Houry et al. 2010), phylogenetically very close to the strain *B. cereus* ATCC 14579 (Lereclus et al. 1982; Sheppard et al. 2013). Examining the β‐galactosidase activity of these strains confirmed that expression of the flagellar gene operon depends on both the presence and the phosphorylation state of CodY (Figure 4C). The P_*fla*_‐directed *lacZ* transcription was high and continuously activated during late exponential growth and early stationary phase in the WT and S215A strains. In accord with the corresponding swimming phenotypes, the S215A mutant exhibited a modest yet statistically significant increase in *fla* promoter activity compared to the wild‐type strain, which suggests that the phosphorylation of CodY plays a negative role in the expression of the *fla* operon. In sharp contrast, no expression was observed in the Δ*codY* and S215D backgrounds. These results agree perfectly with the LC‐MS/MS analysis, which show a drastic reduction in the accumulation of the flagellin BC1659 in the absence of CodY or when CodY is incapable of fulfilling its role (strains codY and S215D, respectively).

These results collectively suggest that (i) CodY‐S215A is functionally competent in vivo, and (ii) CodY phosphorylation plays a regulatory role in modulating the expression of specific target genes. These results corroborate the role of CodY as a positive regulator of the *fla* expression but are quite at odds with our hypothesis after which CodY binding counteracts the transcription of the *fla* operon (see above). Mutation of the CodY box belonging to the *fla* promoter region had no effect when CodY was absent or unable to bind DNA (see expression of the fla*‐*lacZ* fusion in the Δ*codY* and S215D strains, respectively, Figure 4C). Surprinsingly, when the CodY box was inactivated, *lacZ* expression was derepressed during the transition phase in the WT and S215 A backgrounds. As deduced from the localization of the CodY box inbetween the ‐35 and ‐10 promoter boxes and from β‐galactosidase measurements, it is very likely that CodY directly represses the *fla* promoter during the vegetative growth and the transition phase and that another transcriptional inhibitor, itself negatively controlled by CodY, concomitantly represses the expression of the *fla* operon.

### 3.3 Biofilm Formation Is Activated by CodY Phosphorylation

**Figure 5:**
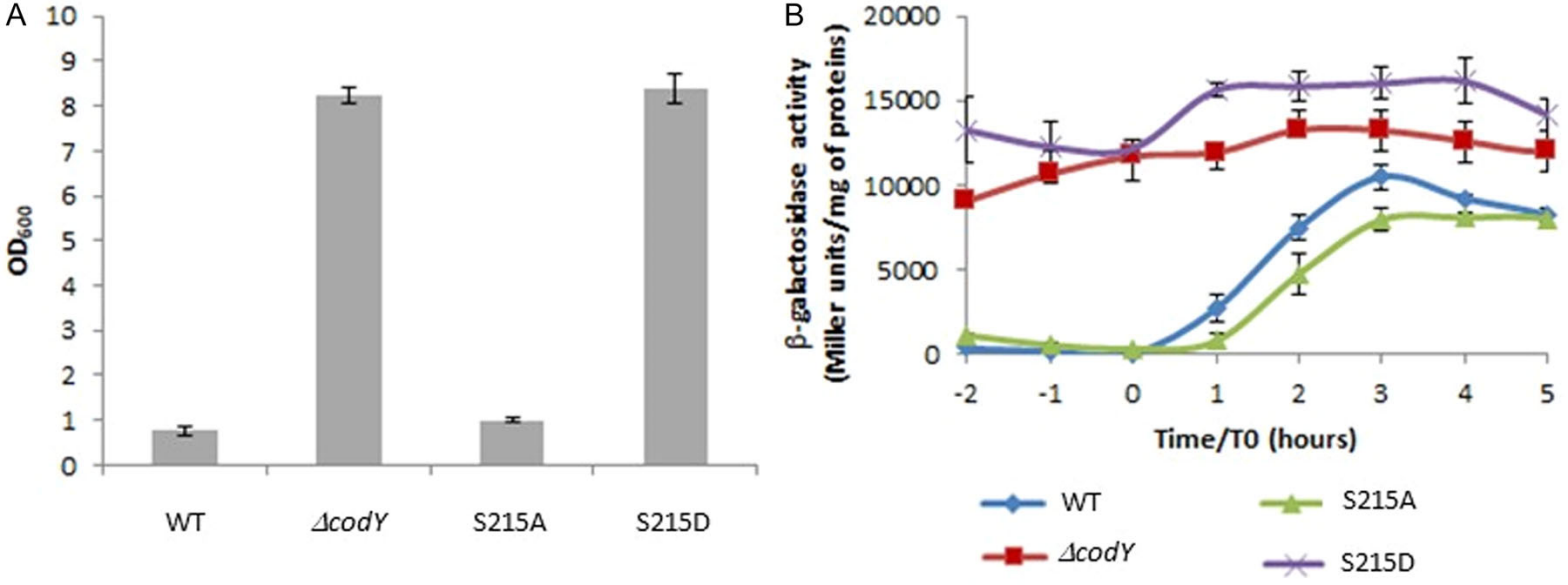
Unphosphorylated CodYrepresses biofilm formation. (A) Production of biofilm in glass tubes. The results are the mean values of for independent experiments and the error bars represent SEM. (B) Expression of a sinI promoter in *B. cereus* codY, S215A and S215D as compared to the WT strain. All strains harbor a transcriptional fusion between the sinI promoter region and the lacZ gene. Cells were grown in LB medium at 37°C under moderate agitation. Samples were harvested every hour before the entry in stationary phase (*t*‐2) to *t*5. The results are the mean values of three independent experiments and the error bars represent SEM.

**Figure 6:**
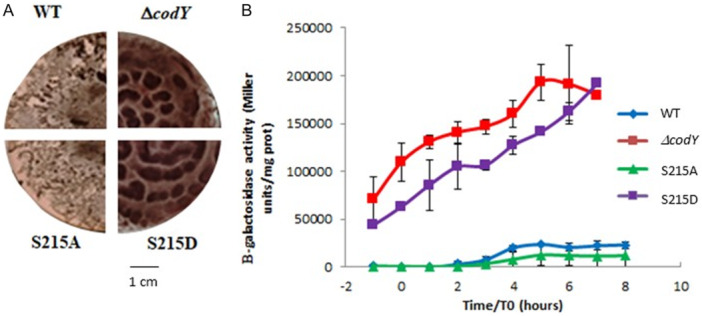
Phosphorylation of CodY leads to amyloïd fibers overexpression in *B. cereus*. (A) Top‐view pictures of floating biofilms of *B. cereus* strains stained with speciﬁc amyloid dye Congo Red after 96 h of growth at 30°C. Strains were grown under static conditions in HCT supplemented with Congo Red (20 µg/ml) and Coomassie Blue (10 µg/ml) in Petri dishes. (B) Expression of β‐galactosidase driven by the calY promoter region in *B. cereus* WT, codY, S215A and S215D strains. Cells were grown in HCT at 30°C. Samples were harvested every 1 h before the entry in stationary phase (*t*‐1) to *t*8. The results are the mean values of three independent experiments and the error bars represent SEM.

Thick biofilm formation at the air–liquid interface is a characteristic feature of *B. cereus* (Auger et al. 2009). Biofilm formation is a differentiation behavior leading to multicellular communities wrapped in self‐producted exopolysaccharide matrix and amyloid fibers bound to a biotic or abiotic surface. The regulatory circuit controlling biofilm synthesis is well characterized in *B. subtilis*. Phosphorylation of the master sporulation regulator Spo0A is central in biofilm accumulation (Hamon and Lazazzera 2001). The transcriptional repressor SinR represses genes required for biofilm formation (Kearns et al. 2005). In biofilm condition, Spo0A promotes the transcription of SinI, a small SinR anti‐repressor, which antagonizes the repression mediated by SinR through protein‐protein interaction (Newman et al. 2013). In *B. cereus* as in *B. subtilis*, SinR negatively controls biofilm formation (Fagerlund et al. 2014; Xu et al. 2017). However, the role of CodY in biofilm formation is controversial: a first study concluded that CodY promotes biofilm formation in *B. cereus* UW101C (Hsueh et al. 2008). By contrast, CodY was shown to repress biofilm formation in strain ATCC14579 (Lindbäck et al. 2012). We checked the capacity of strains WT, Δ*codY*, S215A and S215D to form air‐liquid biofilm in glass tubes (Figure 5A). Total biofilm mass formation was equivalent in strains WT and S215A, while Δ*codY* and S215D strains formed eightfold more biofilm, suggesting that CodY represses biofilm formation. In *B. anthracis*, *sinI* is negatively regulated by CodY (Chateau et al. 2013), and we identified a putative CodY box in the promoter region of *sinI* in ATCC14579 (Supporting Information S1: Figure S2A). We then constructed a *lacZ* transcriptional fusion with the promoter region of *sinI* (plasmid pHT18Z‐*sinI*) and examined its expression (Figure 5B). In both WT and S215A strains, expression of *sinI* was activated during stationary phase from t1 to t3. By contrast, transcription of *sinI* was high and constitutive during vegetative growth and stationary phase in the Δ*codY* and S215D strains. These data are consistent with a model in which CodY represses *sinI* expression up to the transition phase and phosphorylation of S215 contributes to relieve the repression mediated by CodY. Both *prkC* and *ybdM* are expressed during the transition phase occurring between the end of exponential growth and the onset of sporulation (M. Kortebi and S. Poncet, unpublished data). STKs may then phosphorylate CodY and thereby impairs its binding to the *sinI* promoter region. This phosphorylation stage leads, directly and/or indirectly, to activation of the transcription of genes involved in biofilm formation through the SinI‐dependent inactivation of SinR. *B. cereus* biofilms are mainly composed of exopolysaccharides and amyloid fibers, which are polymers of two homologous proteins, TasA and CalY (Caro‐Astorga et al. 2015). In *B. anthracis*, SinR binds to the promoter region of *calY* and represses its transcription (Pflughoeft et al. 2011; Fagerlund et al. 2014). In *B. cereus* ATCC14579, transcription of *calY* is 34.7‐fold derepressed upon deletion of *codY* (Lindbäck et al. 2012). Strains WT, Δ*codY*, S215A and 215D were grown in static conditions in HCT + glucose 0.3% supplemented with Congo Red and Coomassie Blue, an amyloïd dye, in 7 cm‐diameter plates. Similar to the experiments performed in glass tubes, we observed biofilm formation with fibers over production in strains Δ*codY* and S215D (Figure 6A). We then examined the expression of a *calY'‐lacZ* transcriptional fusion in strains grown in HCT at 30°C (Figure 6B). *calY* expression was strongly increased when CodY was absent or unable to bind to DNA. This very high expression of *calY* is likely due to SinI overexpression in the absence of CodY.

### 3.4 Unphosphorylated CodY Is Required for Virulence and Cytotoxicity

**Figure 7:**
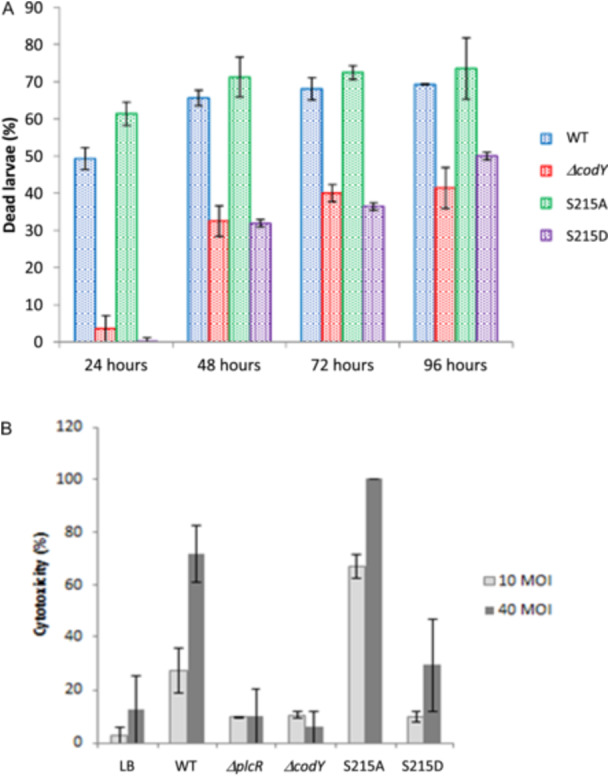
Unphosphorylated CodY is required for *B. cereus* virulence and cytotoxicity. (A) Effect of CodY mutations on virulence after intrahemocoelic injection in Galleria mellonella larvae. Larvae were injected with 6000 mid‐log phase bacteria. Mortality was evaluated after 24, 48, 72 and 96 h of injection. Results are mean values of four independent experiments and errors bars indicate the standard errors of the means. (B) Cytotoxicity to epithelial cells. HeLa cells were infected for 2 h with *B. cereus* culture supernatants at a m.o.i. of 10 or 40. Viable cells remained unstained weather killed cells allowed the trypan blue to stain them. At least 100 cells were counted. Results are mean values of four independent experiments and errors bars indicate the standard errors of the means.

CodY plays an essential role in regulating virulence gene expression within the *Bacillus cereus* group. Virulence of a Δ*codY B. anthracis* strain against mice is known to be attenuated (van Schaik et al. 2009; Château et al. 2011). Similarly, the pathogenicity of a Δ*codY* mutant of *B. cereus* ATCC10987 is attenuated in the insect model *Galleria mellonella* (Frenzel et al. 2012). Phospholipases, enterotoxins, and haemolysins—key secreted virulence factors—are under the direct control of PlcR, a quorum sensor which, after binding with its cognate signaling peptide PapR, activates the expression of target genes (Slamti 2002). The virulence and cytotoxicity of a *plcR* deficient strain is drastically reduced (Gohar et al. 2008; Salamitou et al. 2000). CodY positively post‐translationally regulates the activity of PlcR, essentially through the reimport of PapR (Slamti et al. 2016). To gain deeper insight into the regulatory impact of CodY phosphorylation on virulence, the pathogenicity of strains WT, Δ*codY*, S215A, and S215D was assessed by injecting 6000 vegetative cells into the hemocoel of *G. mellonella* larvae (Figure 7A). At this dose, 50% of the larvae died 24 h after injection of the WT strain. Mortality for the Δ*codY* and S215D strains was significantly reduced: injected larvae began to die only 48 h after injection of Δ*codY and* S215D bacteria, mortality reaching a plateau of about 30%. Interestingly, 24 h post injection, mortality induced by the S215A strain (61.4%) was slightly but significantly higher than the WT strain (49.3%). These results indicate that unphosphorylated CodY is required for pathogenicity in test conditions mimicking opportunistic infection. We also tested the cytotoxic activity of strains WT, Δ*codY*, S215A and S215D to HeLa cells (Figure 7B). At 10 or 40 MOI (Multiplicity of infection), cytotoxicity of Δ*codY and* S215D culture supernatant was lower than that of the WT cells and similar to that in the control (LB) or the non cytotoxic Δ*plcR* cells. By contrast, S215A cells were significantly more cytotoxic than the WT cells. These results clearly indicate that unphosphorylated CodY efficiently promotes the production of extracellular cytotoxic agents (see also Figure 3D).

## 4 Concluding Remarks

Our results strongly indicate that CodY is phosphorylated by Hanks‐type kinases in *B. cereus*. Phosphorylation of S215, a residue crucial for CodY DNA binding activity, completely abolishes its DNA binding. In *S. aureus*, CcpA (catabolite control protein A), a global regulator of the central carbon metabolism and biofilm formation, is also phosphorylated by the Hanks‐type kinase Stk1 in its HTH domain. Similarly to CodY, this phosphorylation abrogates the protein–DNA interaction, leading to the deregulation of CcpA‐repressed genes and operons (Leiba et al. 2012). Stk1 also phosphorylates and inhibits the global regulator VraR in its DNA binding domain, which modulates the cell wall stress stimulon in *S. aureus* (Canova et al. 2014). Similarly, in *B. subtilis*, FatR, which controls fatty acid degradation and stress response, is inactivated via the PtkA‐dependent phosphorylation of Y45, located in the HTH and facing the DNA (Derouiche et al. 2013). Phosphorylation of conserved HTH residues on Ser/Thr or Tyr, as seen in CodY and other examples, likely constitutes a general mechanism for modulating transcriptional regulator activity in bacteria in response to environmental cues. Due to their relaxed specificity and their low efficiency (both in vivo and in vitro), a single Hanks kinase can phosphorylate many substrates and a single substrate can be phosphorylated by several kinases. Thus, inactivation of a single kinase produces a complex pleiotropic phenotype and to study the effect of phosphorylation on one particular substrate, it makes sense to use phosphoablative and phosphomimetic variants of the substrate of interest. This approach has been used in the earlier studies cited above and has proven its worth (Cousin et al. 2013; García García et al. 2018; Bonne Køhler et al. 2020). In *B. cereus* as in *B. subtilis*, the concentration of CodY is not affected by the growth phase, whereas the expression of CodY‐regulated genes varies. Our results show that CodY phosphorylation is an inactivation mechanism which alleviates CodY‐dependent gene control. Consequently, the absence of CodY phosphorylation (S215A strain) reinforces the effect of CodY regulation by prolonging its activity window, at least on virulence, cytoxicity and swimming.

**Figure 8:**
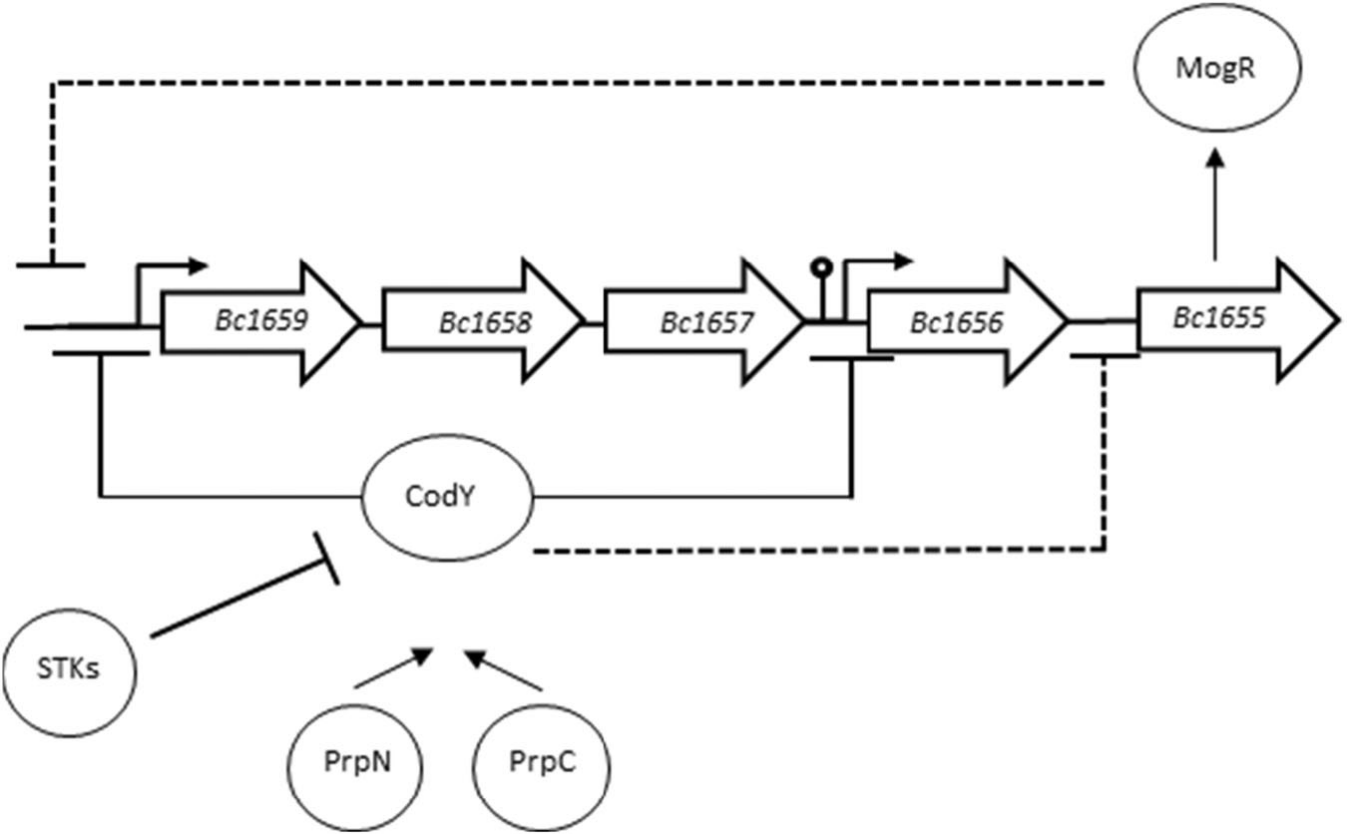
Schematic representation of the proposed CodY‐dependent regulation of the *fla* promoter in ATCC 14579. A model of regulation of the fla and mogR promoters by the combined actions of CodY and MogR. CodY directly represses the transcription of the flagellin genes but also inhibits the expression of the regulatory protein MogR, which in turn inhibits the transcription of the flagellin operon. Inhibitory role of CodY is counteracted by PrkC‐ and YbdM‐dependent phosphorylation on its serine 215 residue. The protein phosphatases PrpN and PrpC dephosphorylate CodY‐S215‐P and help to replenish the active CodY pool.

Previous results showed that CodY acts positively on the expression of the *fla* operon. Our work shows that indeed, CodY directly inhibits the transcription of this operon during the transition phase. To give full account of our results, we propose that CodY also negatively controls, directly or not, the expression of an at yet uncharacterized transcriptional regulator inhibiting the transcription of *fla*. A simplified representation of this hypothetic regulatory loop is given in Figure 8. As a pleiotropic transcriptional regulator repressing motility genes and affecting biofilm formation, stress response, and virulence gene expression, MogR emerges as a suitable candidate (Smith et al. 2020). In *B. anthracis*, a CodY‐binding region was identified in the promoter region of *mogR* (BAS1573), and microarray analysis confirmed that *mogR* is repressed by CodY (Chateau et al. 2013). Similarly, *mogR* (Bc1655) is negatively controled by CodY in *B. cereus* ATCC 14579 (Lindbäck et al. 2012). Further experiments are necessary to confirm or refute this hypothesis.

During infection, bacteria face adversity and progressive nutrition limitations, which are sensed by CodY through its interaction with BCAA and GTP. In *B. cereus* ATCC 14579, Hanks‐type kinase‐dependent phosphorylation of CodY, in response to yet unknown signals seems to contribute to the fine‐tuning of CodY activity in controlling these key physiological processes. Additionally, the serine/threonine phosphatases PrpN and PrpC (BC3861) are probably involved in CodY‐S215‐P dephosphorylation, making the reversible phosphorylation of CodY a key point of control in the life cycle of *B. cereus*.

## 5 Experimental Procedures

### 5.1 Bacterial Strains and Growth Conditions

*Escherichia coli* strain NM522 (Stratagene) was used for cloning purposes, while the *E. coli* strain M15 [pREP4] (Qiagen) was used for protein synthesis. Plasmid DNA for *B. cereus* electroporation was prepared from the Dam^‐^ Dcm^‐^ *E. coli* strain ET12567 (Stratagene, LaJolla, CA, USA). The *B. cereus* ATCC 14579 Δ*codY* mutant is a marker‐less mutant harboring an in frame Δ*codY* deletion which was used as the parental strain to create the *codY* mutant strains used in this study (Lindbäck et al. 2012). Bt407^‐^ chromosomal DNA was used as a template for the PCR reactions which made it possible to clone the *prkC* and the *ybdM* genes into pQE30. Indeed, BLAST analyses showed that CodY, PrkC and YbdM are identical in the two species. *B. cereus* was transformed by electroporation as previously described (Lereclus et al. 1989). *E. coli* strains were grown at 37°C with shaking in Luria Broth (LB) supplemented with ampicillin 100 μg/mL or ampicillin 50 μg/mL + kanamycin 12.5 μg/mL when necessary. *B. cereus* strains were grown at 30 or 37°C with shaking in LB, BHI or HCT, a sporulation medium (Lereclus et al. 1982), supplemented with 10 μg/mL of erythromycin when necessary. t₀ in *B. cereus* cultures represents the moment when the culture transitions from exponential to stationary phase, marked by a reduction in growth rate as the exponential phase concludes.

### 5.2 DNA Manipulations

Chromosomal DNA was extracted from *B. cereus* cells using the Puregene Yeast/Bact Kit B (QIAgen, France). Plasmid DNA was extracted from *E. coli* using QIAprep spin columns (QIAgen, France). Restriction and modification enzymes were used according to the manufacturer (New Biolabs, England). *Pfu* DNA polymerase (Promega) was used for all PCR amplifications. The correct sequence of all the PCR products was confirmed by DNA sequencing.

Plasmid pQE30 (QIAgen) was used for cloning of PCR products for N‐terminal (His)_6_‐tagged protein overproduction. Point mutations *codY*‐S215A and *codY*–S215D were obtained using two partially overlapping mutagenic primers (Supporting Information S1: Table S1). The corresponding PCR products were inserted in the vector pQE‐30 between the BamHI and KpnI sites, giving pQE30‐*ybdM*, pQE30‐*prkC*, pQE30‐*codY, ‐codY*‐S215A and ‐*codY*–S215D, respectively (Table 1 and Supporting Information S1: Table S1).

**Table 1:** Plasmids used in this study.

Name	Relevant features	Reference
pQE30‐*codY*	*codY* was PCR‐amplified from the chromosome of strain ATCC14579 using primer pair SAT31 + SAT32 and cloned between the BamHI and the KpnI restriction sites of pQE‐30	This study
pQE30‐*codY*‐S215A	The point mutation *codY*‐S215A was amplified by PCR from the chromosome of strain ATCC14579 using primer pair SAT31 + SAT45 and SAT46 + SAT32 followed by overlap extension PCR using the two overlapping PCR products and primer pair SAT31 + SAT32 and cloned between the BamHI and KpnI restriction sites of pQE‐30	This study
pQE30‐*codY*‐S215D	Same as pQE‐30‐codY‐S215A but with primer pairs SAT31 + SAT47 and SAT48 + SAT32	This study
pQE30‐*prkC*	The 5' part of *prkC*, encoding the cytosolic kinase domain of PrkC, was PCR‐amplified from the chromosome of strain Bt407^‐^ using primer pair SAT7 + SAT8 and cloned between the BamHI and the KpnI restriction sites of pQE‐30	This study
pQE30‐*ybdM*	*ybdM* was PCR‐amplified from the chromosome of strain Bt407^‐^ using primer pair SAT5 + SAT6 and cloned between the BamHI and the KpnI restriction sites of pQE‐30	This study
pMAD*−codY‐*S215A	*codY* and flanking regions were amplified by PCR from the chromosome of ATCC14579 using primer pairs SAT108 + SAT45 and SAT46 + SAT109 followed by overlap extension PCR using the two overlapping PCR products and primer pair SAT108 + SAT109. The resulting 2800 bp replicon was cloned between the KpnI and BamHI restriction sites of the thermosensitive plasmid pMAD.	This study
pMAD*−codY‐*S215D	*codY* and flanking regions were amplified by PCR from the chromosome of ATCC14579 using primer pairs SAT108 + SAT47 and SAT48 + SAT109 followed by overlap extension PCR using the two overlapping PCR products and primer pair SAT108 + SAT109. The resulting 2800 bp replicon was cloned between the KpnI and BamHI restriction sites of the thermosensitive plasmid pMAD.	This study
pHT18Z‐*fla*	*fla* promoter region was amplified by PCR from the chromosome of ATCC14579 using primer pair SAT205 + SAT206, and cloned between the PstI and BamHI restriction sites of pHT304‐18Z	This study
pHT18Z‐fla*	*fla* promoter region was amplified by PCR from the chromosome of ATCC14579 using primer pair SAT205 + SAT204 and SAT203 + SAT206 followed by overlap extension PCR using the two overlapping PCR products and primer pair SAT205 + SAT206. The resulting PCR product was cloned between the PstI and XbaI restriction sites of pHT304‐18Z.	This study
pHT18Z‐*sinI*	*sinI* promoter region was amplified by PCR from the chromosome of ATCC14579 using primer pairs SAT207 + SAT208, and cloned between the PstI and XbaI restriction sites of pHT304‐18Z	This study
pHT18Z‐*calY*	*calY* promoter region was amplified by PCR from the chromosome of ATCC14579 using primer pairs SAT209 + SAT210, and cloned between the PstI and XbaI restriction sites of pHT304‐18Z	This study

pHT18Z‐*rpsB*, pHT18Z‐*fla*, pHT18Z‐fla*, pHT18Z‐*sinI*, and pHT18Z‐*calY* (Table 1 and Supporting Information S1: Table S1) were obtained by inserting the corresponding promoter regions between the XbaI and PstI cloning sites of pHT304–18Z (Agaisse and Lereclus 1994). The resulting plasmids were then transferred into *B. cereus* by electroporation.

pMAD is a thermosensitive plasmid allowing allelic exchanges *in B. cereus* (Arnaud et al. 2004). Plasmids pMAD*−codY‐*S215A and pMAD*−codY‐*S215D were used to introduce *codY*‐S215 or –S215D, respectively, in strain ATCC14579 Δ*codY* (See Table 1 and Supporting Information S1: Table S1). Strains ATCC14579 *codY*‐S215A and –S215D were constructed as follows. The thermosensitive pMad‐*codY‐*S215A and –S215D were introduced by electroporation in strain ATCC14579 Δ*codY*, and were integrated in the chromosome following a single recombination event. After verification of the resulting Erm^R^ strains ATCC14579Δ*codY*::pMad‐*codY*‐S215A and –S215D cells by PCR, the second recombination event was allowed to proceed, leading to Erm^S^ strains ATCC14579 *codY*‐S215A and *codY*‐S2515D. Each step was verified by PCR amplification and sequence, using oligonucleotides hybridizing upstream and downstream from the *codY* gene (VcodYBc1 and VcoYBc2, see Supporting Information S1: Table S1). *B. cereus* strains ATCC14579, ATCC14579Δ*codY*, ATCC14579 Δ*codY::codY*‐S215A and ATCC14579Δ*codY::codY*‐S215D will further be referred as WT, *codY*, S215A and S215D strains, respectively.

### 5.3 Synthesis and Purification of Affinity‐Tagged Proteins and Protein Phosphorylation Assays

Synthesis and purification of N‐Terminal 6xHis‐tagged proteins and in vitro phosphorylation assays were performed as described previously (Poncet et al. 2009). Briefly, overproduced proteins were recovered from *E. coli* crude extracts after induction by IPTG (1 mM, 3 h at 37°C), then purified on a Ni‐NTA affinity column (Qiagen) and desalted on pD10 Columns (GE‐Healthcare) following the manufacturer recommandations. Protein concentration was quantified by the Bradford assay (BioRad), and proteins were stored at −20°C in a glycerol‐containing buffer. In vitro phosphorylation assays were performed in the presence of 50 µM ATP, containing 20 µCi mmol^−1^ [γ‐^32^P]‐ATP, 1 h at 37°C. Protein concentrations used were as follows: 1 μM PrkC or YbdM, 4 μM CodY or CodY‐S215A. Phosphorylated proteins were visualized via autoradiography with a FUJI phosphoimager.

### 5.4 Gel Filtration Assay

The gel filtration assays were performed with an Akta Purifier HPLC system, using a Superdex 75 h 30/4 column (GE Healthcare) in isocratic conditions. The column was equilibrated with PBS 1x and calibrated using a low molecular weight GE Healthcare calibration kit (Supporting Information S1: Figure S1A). 75 μL of purified CodY WT, ‐S215A and ‐S215D ( ≈ 150 μg) were loaded individually on the column and eluted with PBS 1x at a flow of 0.5 mL min^−1^. Absorbance at 280 nm was used to monitor protein content in the eluate in real time. The experiment was repeated twice and one representative result is shown.

### 5.5 Circular Dichroism

Circular dichroism (CD) spectra were recorded with a Jasco J‐815 spectropolarimeter featuring a temperature control system based on Peltier technology (Model PTC‐423S). CD measurements (185–270 nm) were carried out in Tris 50 mM pH 7.4 NaCl, 100 mM at 20°C by using a 0.1 cm optical path length cell. Spectra were recorded with parameters of 4 s time constant, 2 nm bandwidth, and 20 nm/min scan rate, averaged over a minimum of three scans, and baseline‐corrected by buffer subtraction.

### 5.6 Electrophoretic Mobility Shift Assays

The electrophoretic mobility shift assay were performed in a reaction buffer containing 25 mM Tris‐HCl, pH 7.5, 50 mM NaCl, 5% glycerol, 1 mM DTT and 10 mM MgCl_2_. GTP 4 mM and BCAA 5 mM (mix of leucine, isoleucine and valine) were added when necessary. Concentrations of protein and DNA in the assays are indicated in the figure legend. The promoter region of the *B. cereus* flagellin operon (gene annotation Bc 1659‐1658‐1657), including the potential CodY box, was PCR amplified (oligonucleotides SAT201 + SAT202) and used as substrate for DNA binding. The same region carrying a mutated CodY box was obtained by overlap extension PCR using the same oligonucleotides combined with the mutagenic primers SAT203 and SAT204 (Supporting Information S1: Table S1). Reactions mixtures were incubated at room temperature for 20 min and loaded directly onto a 6% polyacrylamide gel (50 mM Tris‐HCl, pH 8.5; 400 mM glycine; 2.5% glycerol) for electrophoresis. Signals were revealed by ethidium bromide staining. All experiments were performed in duplicate and one representative result is shown.

### 5.7 Western Blot

For each strain, cells were disrupted with glass beads (212– 300 mm; Sigma) in a Fast‐Prep 24 (MP Biomedical), and cell extracts were obtained after centrifugation. Aliquots corresponding to 10 μg of cytosolic proteins were mixed with a 5X buffer [300 mM Tris (pH 6.8), 50% glycerol, 10% SDS, 5% β‐mercaptoethanol, bromophenol blue], heated 5 min at 100°C and resolved on 12% SDS‐PAGE gels, which were transferred to nitrocellulose membranes using the Iblot II system (Invitrogen). Membranes were incubated for 2 h with blocking buffer [8% (wt/vol) nonfat milk in PBS], washed three times with PBST (PBS containing 0.1% Tween 20) for 10 min and incubated with an anti‐CodY rabbit serum diluted 1:2000‐fold for 2 h at room temperature. Membranes were washed three times with PBST and then incubated with secondary anti‐rabbit peroxidase‐conjugated antibodies (Sigma Aldrich, at 1:10,000) for 2 h. Membranes were washed three times with PBST and incubated with ECL peroxidase substrate as recommended by the manufacturer (Thermofischer).

### 5.8 Protein Identification by Mass Spectrometry

Samples were taken at 2 h after t0 from WT, *codY*, S215A, and S215D cultures cultivated in BHI at 37°C. Proteins were resuspended in Laemmli buffer and loaded on SDS‐PAGE gels. Gel bands were excised and proteins were reduced, alkylated, and digested in‐gel with trypsin overnight, following a protocol similar to that described by Millán‐Oropeza et al. (2022). Peptides were subsequently extracted with 5% formic acid in 50% acetonitrile (v/v) before mass spectrometry analysis. HPLC was performed on a Dionex Ultimate 3000 RSLC system with a 4‐μL sample loaded at 20 μL/min onto a C18 PepMap 100 precolumn, followed by separation on a 75 μm × 150 mm PepMap C18 column using a linear gradient from 0% to 36% solvent B over 18 min at 300 nL/min. The eluted peptides were analyzed online by an Orbitrap Lumos Fusion Tribrid mass spectrometer with a nanoelectrospray interface and ionization potential of 1.3 kV as previously described (Millán‐Oropeza et al. 2022). The data were converted into mzXML format using MS convert (ProteoWizard, version 3.0.8934). Database searches were performed using Database *Bacillus cereus* ATCC14579 (5337 entries, version November 2024). i2MassChroQ software was used (version 1.0.18 http://pappso.inrae.fr/) with one possible miss cleavage. Carboxyamidomethylation of cysteine residues was set as a fixed modification, while oxidation of methionine residues was considered a variable modification. Precursor and fragment mass tolerance were set to 10 ppm and 0.5 Da, respectively. Data filtering was applied using the following criteria: peptide E‐value < 0.05, protein log(E‐value) < –2.6, and a minimum of two peptides identified per protein.

### 5.9 Motility and Biofilm Assays

The swimming capacity of *B. cereus* strains was determined on LB soft (0.2%) agar plates. Strains were grown in LB medium at 37°C until the culture reached an OD_600_ of 1. For each culture, a 5 μL drop was spotted on a 0.2% agar plate and incubated overnight at 30°C. Experiments were performed in duplicate and one representative result is shown. The expansion rate was calculated by measuring the colony diameter as a function of the incubation time at room temperature. Experiments were performed in duplicate with four independent clones for each strain.

The ability of the strains to form biofilm in glass tubes was tested as previously described (El‐Khoury et al. 2016). Cultures in the mid‐exponential phase (OD₆₀₀ ≈ 1) were diluted to an OD₆₀₀ of 0.01 in 2 mL of HCT medium and incubated statically at 30°C for 48 h. After incubation, the culture medium was gently removed using a Pasteur pipette. The remaining biofilm was resuspended by thorough vortexing in 1 mL of PBS, and the OD₆₀₀ was measured to quantify biofilm biomass. Staining of *B. cereus* pellicles with the amyloid dye Congo Red was performed by growing the cells 4 days at room temperature in Petri dishes 7 cm in diameter in HCT medium supplemented with Congo Red and Coomassie Brilliant Blue G at final concentrations of 20 and 10 μg/mL, respectively.

### 5.10 β‐Galactosidase Assay

β‐Galactosidase activity was measured as previously described with an incubation temperature set to 28°C (Perchat et al. 2011). Mean values of at least three independent assays are presented.

### 5.11 In Vivo Experiments

Intrahemocelic injection experiments with *Galleria mellonella* larvae were carried out as previously described (Bouillaut et al. 2005). Last‐instar larvae were injected with 10 μL of mid‐log phase bacteria suspended in PBS, using a microinjector (Buckard Scientific, UK.). The same dose of *B. cereus* WT, *codY*, S215A and S215D was used (6000 vegetative cells/larvae). Four independent experiments were carried out, each including three samples of 20 larvae for each strain. Infected larvae were kept at 30°C and mortality was recorded 24, 48, 72, and 96 h postinjection. For cytotoxicity assays, HeLa cells were infected with *B. cereus* culture supernatant at a m.o.i. of 10 and 40 for 2 h (Ramarao and Lereclus 2006). Trypan blue was then added, allowing to distinguish viable cells (unstained) from killed cells (stained). Results are mean values of for independent experiments.

## Author Contributions

**Mounia Kortebi:** conceptualization, investigation, visualization, writing – original draft. **Céline Henry:** investigation, visualization, writing review. **Christophe Buisson:** investigation. **Christelle Lemy:** investigation. **Michel Gohar:** methodology, writing – review. **Didier Lereclus:** funding acquisition, writing – review. **Ivan Mijakovic:** writing – review. **Sandrine Poncet:** conceptualization, data curation, formal analysis, funding acquisition, investigation, methodology, project administration, resources, supervision, validation, visualization, writing – original draft, writing – review and editing.

## Ethics Statement

The authors have nothing to report.

## Conflicts of Interest

The authors declare no conflicts of interest.

## Supporting information

Revised supporting informations.

## Data Availability

All the data generated by these studies is reported in this paper and its supporting data file.
