# Supplementary material for: Impact of Hanks Kinase‐Dependent Phosphorylation of CodY on the Physiology and Virulence in Bacillus cereus
Source: Microbiologyopen. 2025 Nov 4;14(6):e70103. doi: 10.1002/mbo3.70103 (PMC12583987; doi:10.1002/mbo3.70103)
Supplement: Supplementary file 1 — Revised supporting informations. [file MBO3-14-e70103-s001.pptx]

## Slide 1
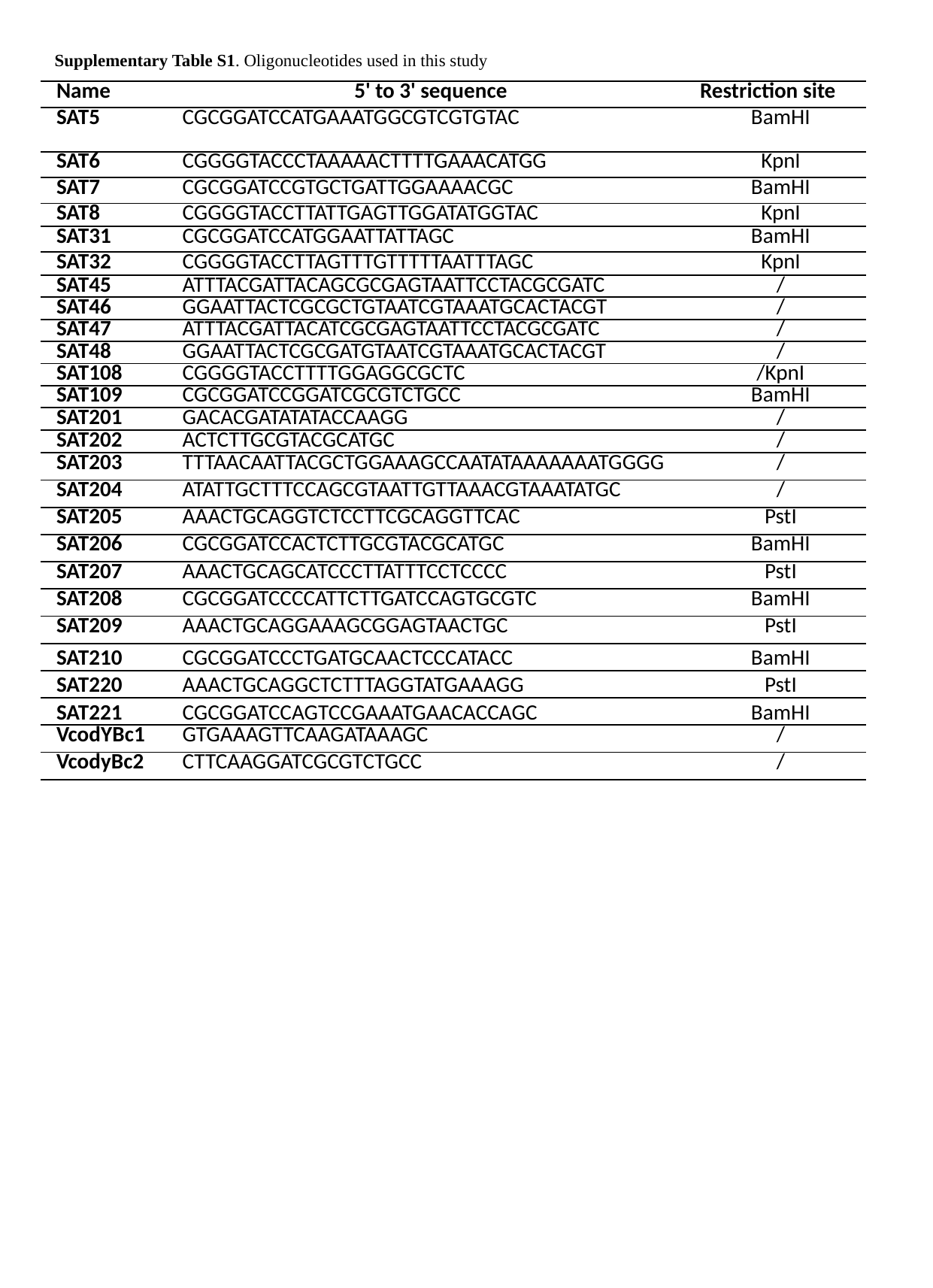

Supplementary Table S1. Oligonucleotides used in this study
| Name | 5' to 3' sequence | Restriction site |
| --- | --- | --- |
| SAT5 | CGCGGATCCATGAAATGGCGTCGTGTAC | BamHI |
| SAT6 | CGGGGTACCCTAAAAACTTTTGAAACATGG | KpnI |
| SAT7 | CGCGGATCCGTGCTGATTGGAAAACGC | BamHI |
| SAT8 | CGGGGTACCTTATTGAGTTGGATATGGTAC | KpnI |
| SAT31 | CGCGGATCCATGGAATTATTAGC | BamHI |
| SAT32 | CGGGGTACCTTAGTTTGTTTTTAATTTAGC | KpnI |
| SAT45 | ATTTACGATTACAGCGCGAGTAATTCCTACGCGATC | / |
| SAT46 | GGAATTACTCGCGCTGTAATCGTAAATGCACTACGT | / |
| SAT47 | ATTTACGATTACATCGCGAGTAATTCCTACGCGATC | / |
| SAT48 | GGAATTACTCGCGATGTAATCGTAAATGCACTACGT | / |
| SAT108 | CGGGGTACCTTTTGGAGGCGCTC | /KpnI |
| SAT109 | CGCGGATCCGGATCGCGTCTGCC | BamHI |
| SAT201 | GACACGATATATACCAAGG | / |
| SAT202 | ACTCTTGCGTACGCATGC | / |
| SAT203 | TTTAACAATTACGCTGGAAAGCCAATATAAAAAAATGGGG | / |
| SAT204 | ATATTGCTTTCCAGCGTAATTGTTAAACGTAAATATGC | / |
| SAT205 | AAACTGCAGGTCTCCTTCGCAGGTTCAC | PstI |
| SAT206 | CGCGGATCCACTCTTGCGTACGCATGC | BamHI |
| SAT207 | AAACTGCAGCATCCCTTATTTCCTCCCC | PstI |
| SAT208 | CGCGGATCCCCATTCTTGATCCAGTGCGTC | BamHI |
| SAT209 | AAACTGCAGGAAAGCGGAGTAACTGC | PstI |
| SAT210 | CGCGGATCCCTGATGCAACTCCCATACC | BamHI |
| SAT220 | AAACTGCAGGCTCTTTAGGTATGAAAGG | PstI |
| SAT221 | CGCGGATCCAGTCCGAAATGAACACCAGC | BamHI |
| VcodYBc1 | GTGAAAGTTCAAGATAAAGC | / |
| VcodyBc2 | CTTCAAGGATCGCGTCTGCC | / |

## Slide 2
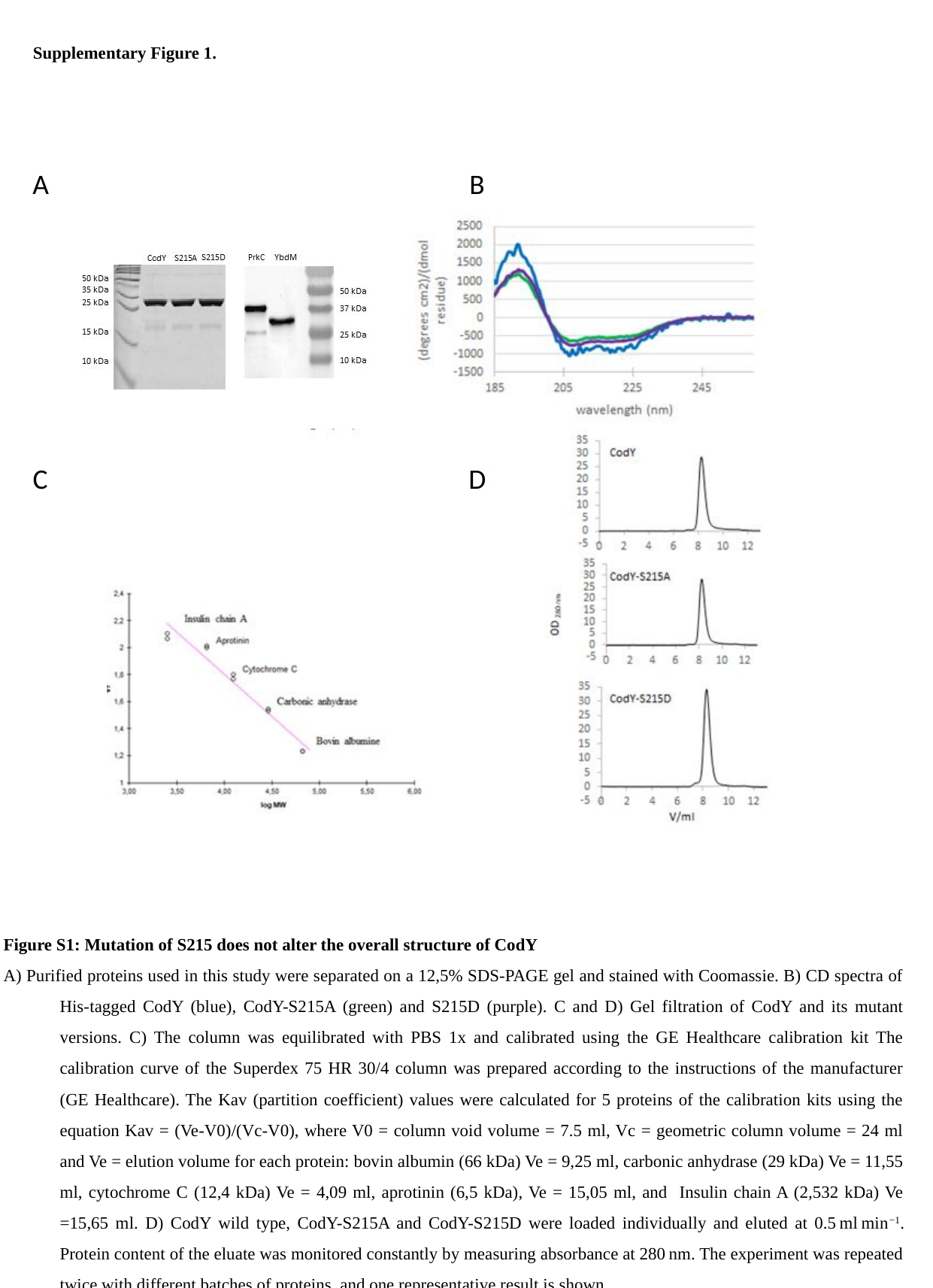

Supplementary Figure 1.
A
B
C
D
Figure S1: Mutation of S215 does not alter the overall structure of CodY
A) Purified proteins used in this study were separated on a 12,5% SDS-PAGE gel and stained with Coomassie. B) CD spectra of His-tagged CodY (blue), CodY-S215A (green) and S215D (purple). C and D) Gel filtration of CodY and its mutant versions. C) The column was equilibrated with PBS 1x and calibrated using the GE Healthcare calibration kit The calibration curve of the Superdex 75 HR 30/4 column was prepared according to the instructions of the manufacturer (GE Healthcare). The Kav (partition coefficient) values were calculated for 5 proteins of the calibration kits using the equation Kav = (Ve-V0)/(Vc-V0), where V0 = column void volume = 7.5 ml, Vc = geometric column volume = 24 ml and Ve = elution volume for each protein: bovin albumin (66 kDa) Ve = 9,25 ml, carbonic anhydrase (29 kDa) Ve = 11,55 ml, cytochrome C (12,4 kDa) Ve = 4,09 ml, aprotinin (6,5 kDa), Ve = 15,05 ml, and Insulin chain A (2,532 kDa) Ve =15,65 ml. D) CodY wild type, CodY-S215A and CodY-S215D were loaded individually and eluted at 0.5 ml min−1. Protein content of the eluate was monitored constantly by measuring absorbance at 280 nm. The experiment was repeated twice with different batches of proteins, and one representative result is shown

## Slide 3
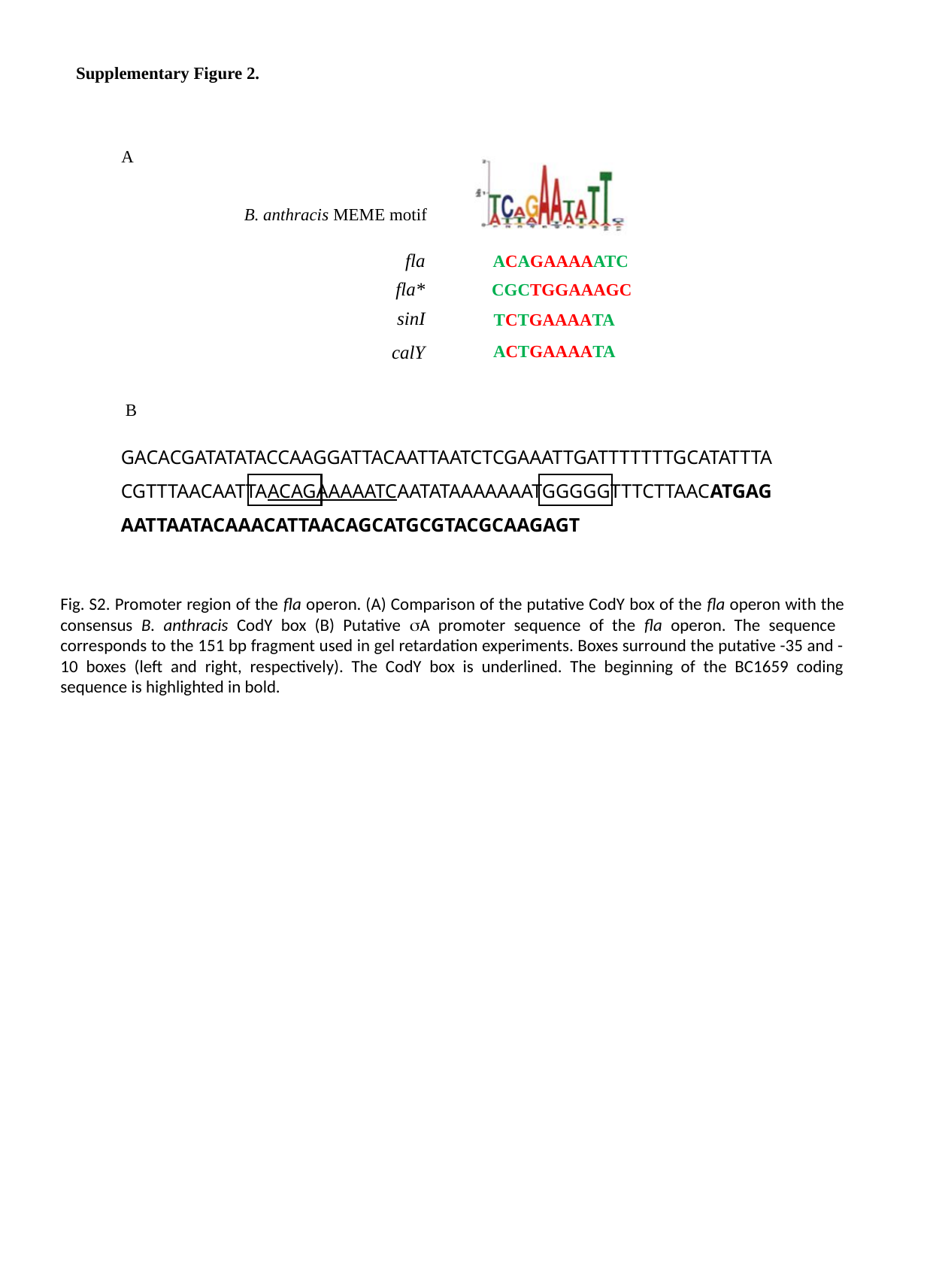

Supplementary Figure 2.
A
 B. anthracis MEME motif
fla
ACAGAAAAATC
fla*
CGCTGGAAAGC
sinI
TCTGAAAATA
calY
ACTGAAAATA
B
GACACGATATATACCAAGGATTACAATTAATCTCGAAATTGATTTTTTTGCATATTTACGTTTAACAATTAACAGAAAAATCAATATAAAAAAATGGGGGTTTCTTAACATGAGAATTAATACAAACATTAACAGCATGCGTACGCAAGAGT
Fig. S2. Promoter region of the fla operon. (A) Comparison of the putative CodY box of the fla operon with the consensus B. anthracis CodY box (B) Putative sA promoter sequence of the fla operon. The sequence corresponds to the 151 bp fragment used in gel retardation experiments. Boxes surround the putative -35 and -10 boxes (left and right, respectively). The CodY box is underlined. The beginning of the BC1659 coding sequence is highlighted in bold.

## Slide 4
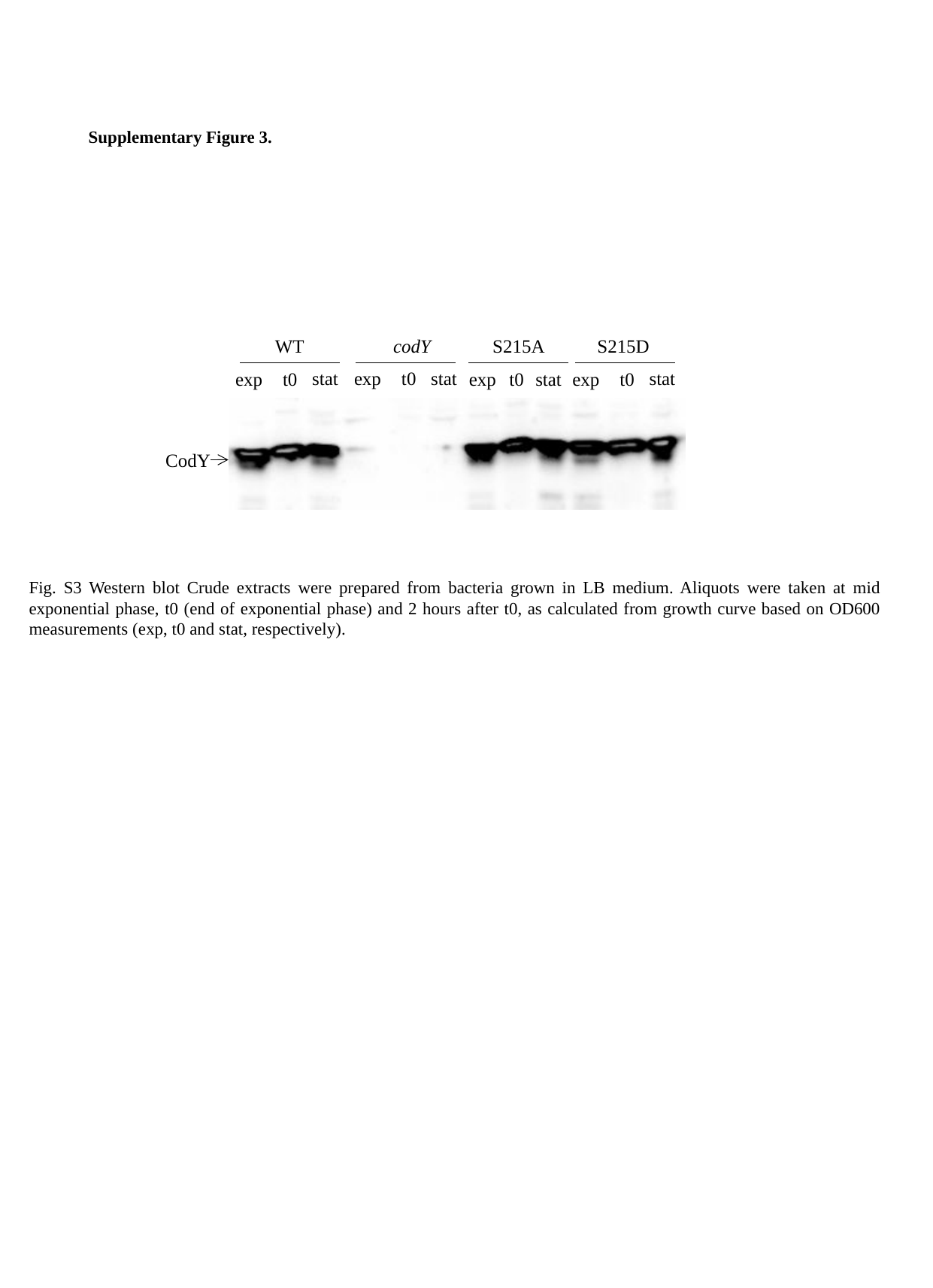

Supplementary Figure 3.
WT
codY
S215A
S215D
stat
exp
t0
stat
stat
stat
exp
t0
exp
t0
exp
t0
CodY
Fig. S3 Western blot Crude extracts were prepared from bacteria grown in LB medium. Aliquots were taken at mid exponential phase, t0 (end of exponential phase) and 2 hours after t0, as calculated from growth curve based on OD600 measurements (exp, t0 and stat, respectively).
